# Rate of Ipsilateral Chronic Limb-Threatening Ischemia (CLTI) After Kidney Transplantation: A Retrospective Single-Center Study

**DOI:** 10.7759/cureus.25455

**Published:** 2022-05-29

**Authors:** Abdul Kader Natour, Ziad Al Adas, Timothy Nypaver, Alexander Shepard, Mitchell Weaver, Lauren Malinzak, Anita Patel, Loay Kabbani

**Affiliations:** 1 Vascular Surgery, Henry Ford Health System, Detroit, USA; 2 Transplant Surgery, Henry Ford Health System, Detroit, USA; 3 Transplant Nephrology, Henry Ford Health System, Detroit, USA

**Keywords:** lower extremity revascularization, end-stage renal disease, peripheral artery disease, kidney transplantation, chronic limb threatening ischemia

## Abstract

Objective: To analyze whether the rate of lower extremity (LE) ischemia is higher on the ipsilateral side after kidney transplantation.

Methods: Our institutional transplant database was retrospectively queried for all patients who received a kidney transplant and underwent subsequent LE revascularization or major limb amputations between January 2004 and July 2020. The one-sample binomial test was used to test whether the LE ipsilateral to the transplanted kidney was at higher risk of peripheral arterial disease (PAD) complications necessitating intervention (major amputation or revascularization).

Results: There were 1,964 patients who received a kidney transplant during the study period. Of these, 51 patients (3%) had subsequent LE arterial revascularizations or major amputations. The mean age was 58 ± 10 years, and 37 patients (73%) were male. A total of 33 patients had ipsilateral LE vascular interventions (26 major amputations and seven revascularizations) while 18 patients had contralateral vascular interventions (14 major amputations and four revascularizations) (P = 0.049). The average interval between transplantation and subsequent vascular intervention was 52 months for the ipsilateral intervention group and 41 months for the contralateral intervention group (P = 0.33).

Conclusions: In patients who received kidney transplantation and required subsequent LE surgical intervention, we observed an association between the side of transplantation and the risk of future ipsilateral LE arterial insufficiency. Further studies are needed to determine the etiology of this association.

## Introduction

End-stage renal disease (ESRD) is a global health problem, and despite increased public health awareness and preventive measures, the prevalence of ESRD is increasing annually [1]. ESRD and contributing conditions, such as diabetes and hypertension, are known risk factors for atherosclerosis and peripheral arterial disease (PAD). Although ESRD has been shown to be a negative predictor for both limb salvage and survival in PAD patients, the effects of kidney transplantation on PAD have not been extensively studied [2-6]. Kidney transplantation improves overall patient survival. However, it is an invasive arterial procedure, and vascular complications are a recognized and serious problem after transplant surgery [7]. In addition, patients undergoing kidney transplantation are likely at greater risk for the development of PAD when compared to the general population, owing to the immunosuppressive state and the high prevalence of risk factors for atherosclerosis in this patient population, such as hypertension, hyperlipidemia, and diabetes [8]. The manifestation of PAD in the transplant population is of particular concern, since it might lead to deleterious effects on the limb and patient survival due to impaired wound healing and increased risk of infection associated with the immunosuppressive state.

Kidney transplant candidates frequently undergo preoperative peripheral vascular evaluation (mainly iliac artery assessment) in the aim of preventing post-transplant vascular complications [9,10]. Cases of acute ipsilateral limb ischemia have been reported after kidney transplantation, but these are usually presumed to be technical in nature [7,11,12]. The few studies addressing the association of kidney transplantation with lower extremity (LE) PAD have produced conflicting results [7-9,13-15]. Moreover, a theoretical risk of blood diversion from a previously under-perfused leg after kidney transplantation has been a major clinical concern for this population [9,10]. Nonetheless, this “blood steal” phenomenon is not well explored in the literature, and it remains unclear whether the side of the transplanted kidney is associated with the side of PAD development. We hypothesized that ischemic events in the ipsilateral LE would occur more frequently than contralateral LE ischemic events after kidney transplantation.

## Materials and methods

This single-institution retrospective record review study was approved by the Henry Ford Health System Institutional Review Board - Edsel Board (No. 11971) before its initiation, and the need for informed consent was waived.

Patient population

We retrospectively queried the transplant database at our quaternary care center for all patients who had received kidney transplantation between January 2004 and July 2020. This list was cross-referenced with a vascular surgery database of patients who underwent major LE amputations (below or above knee) or LE revascularization procedures at our institution during the same time period. Patients who had a LE intervention prior to the kidney transplant or within 30 days post transplant and those who had bilateral kidney transplants or concomitant pancreatic transplants were excluded from the study. Patients who had technical errors at the anastomotic site that had caused stenosis as confirmed by postoperative duplex ultrasound were excluded from the study as well. Only patients diagnosed with PAD prior to the vascular procedure by performing ankle-brachial index or computed tomographic angiography were included in the study. For patients who underwent both a revascularization and a major amputation after the kidney transplant, the intervention that occurred first was counted. The study endpoint was the time of the vascular intervention. Patient demographics (age, sex, and race), comorbidities (hypertension, type 2 diabetes, chronic obstructive pulmonary disease, dyslipidemia, coronary artery disease, pre-transplant PAD, history of stroke, and tobacco use), medications (aspirin, beta-blockers, statins, and immunosuppressants), and surgical details (side of the LE intervention and side of the kidney transplant) were collected from the electronic medical record.

Statistical analysis

The main question addressed in this study was whether the LE ipsilateral to the transplanted kidney was at higher risk of PAD complications necessitating intervention (major amputation or revascularization). The one sample binomial test was used to test our hypothesis: it assumes that the risk of an ipsilateral LE intervention should be equal to the risk of a contralateral LE intervention in the sample. We divided our patient sample into two groups: patients who received an ipsilateral LE intervention (major amputation or revascularization) and patients who received a contralateral LE intervention after the kidney transplant. The Student’s t test was used to compare the time from the kidney transplant to the subsequent major amputation or revascularization between the groups.

## Results

Patient sample and characteristics

**Figure 1:**
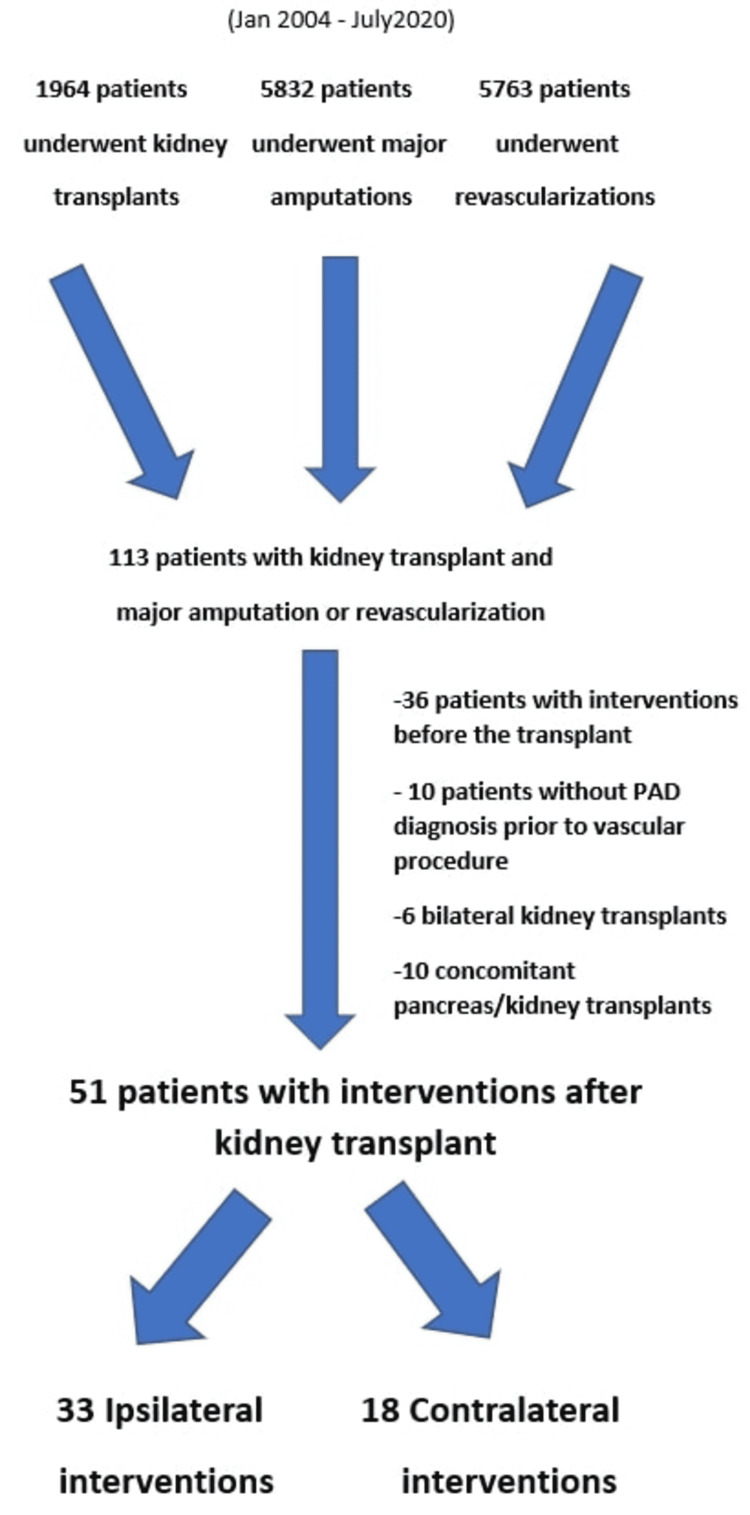
Patient exclusion algorithm PAD: peripheral arterial disease

From 2004 to 2020, 1,964 patients received a kidney transplant at our institution. After matching these patients with 5,832 patients who underwent a LE major amputation and 5,763 patients who underwent open or endovascular LE revascularizations during the same time period, 113 patients were identified who had received a kidney transplant and had undergone a major amputation or a revascularization procedure during this 16-year interval. There were 36 patients who received an intervention prior to the kidney transplant, and 26 patients were excluded from the study, including six patients with bilateral kidney transplants, 10 patients with concomitant kidney/pancreatic transplants, and 10 patients who underwent vascular procedure for reasons other than PAD. The study sample consisted of 51 patients who received a single-kidney transplant and who underwent a subsequent major amputation or revascularization for PAD (Figure 1).

The incidence of chronic limb-threatening ischemia (CLTI) in the study group was 2.6% (n = 51). Of the 51 patients, the mean age was 58 ± 10 years, and men comprised 73% of the cohort (n = 37). In the cohort, 28 (60%) were African American, 17 (36%) were White, 48 (94%) had hypertension, 44 (86%) had type 2 diabetes, 27 (53%) had coronary artery disease, and four (8%) were current smokers. Most of the patients (90%; n = 46) were on some form of immunosuppressive therapy. While 78% (n = 36) of the patients had prednisone as part of their immunosuppressive regimen, 89% (n = 41) had tacrolimus and 70% (n = 32) had mycophenolate mofetil. At presentation with a PAD complication, 38 (75%) patients were on an antiplatelet agent and 33 (65%) were on a statin (Table 1). At the end of the study period, most of the patients (90%; n = 46) had a functional kidney transplant, and five patients (10%) had graft failure due to acute or chronic autoimmune rejection with no graft nephrectomy performed.

**Table 1:** General description of the cohort and comparison of the baseline characteristics, comorbidities, and medications between patients that had ipsilateral or contralateral LE intervention after renal transplant LE: lower extremity; PAD: peripheral arterial disease

Variable	LE Intervention Post-Renal Transplant (n = 51)	Ipsilateral Intervention (n = 33)	Contralateral Intervention (n = 18)	P value
Demographics				
Age, years, mean ± SD	57.6 ± 9.7	57.6 ± 10.2	57.8 ± 9.1	0.92
Body mass index, kg/m^2^, mean ± SD	30.8 ± 5.0	30.8 ± 5.0	30.8 ± 5.0	0.99
Gender, N (%)				0.20
Female	14 (27.0)	11 (33.3)	3 (16.7)	
Male	37 (73.0)	22 (66.7)	15 (71.4)	
Race, N (%)				0.29
African American	28 (59.5)	15 (51.7)	13 (72.2)	
Asian	2 (4.3)	1 (3.4)	1 (5.6)	
White	17 (36.2)	13 (44.8)	4 (22.2)	
Comorbidities, N (%)				
Hypertension	48 (94.1)	31 (93.9)	17 (94.4)	0.94
Type 2 diabetes	44 (86.3)	28 (84.8)	16 (88.9)	0.70
Coronary artery disease	27 (52.9)	16 (48.5)	11 (61.1)	0.40
Cerebrovascular accident	5 (9.8)	4 (12.1)	1 (5.6)	0.45
Chronic obstructive pulmonary disease	3 (5.9)	2 (6.1)	1 (5.6)	0.94
Dyslipidemia	29 (56.9)	22 (66.7)	7 (38.9)	0.06
Pre-transplant PAD	6 (11.8)	4 (12.5)	2 (11.2)	0.88
Anemia (hemoglobin <12 g/dL)	33 (66.0)	21 (65.6)	12 (66.7)	0.94
Smoker, N (%)				0.50
Never	27 (53.0)	19 (57.6)	8 (44.4)	
Current	4 (7.8)	3 (9.1)	1 (5.6)	
Previous	20 (39.2)	11 (33.3)	9 (50.0)	
Medications, N (%)				
Aspirin (or other antiplatelets)	38 (74.5)	26 (78.8)	12 (66.7)	0.34
Angiotensin-converting enzyme inhibitors	10 (19.6)	5 (15.2)	5 (27.8)	0.28
Beta-blockers	39 (76.5)	26 (78.8)	13 (72.2)	0.60
Statins	33 (64.7)	21 (63.6)	12 (66.7)	0.83
Immunosuppressive therapy	46 (90.2)	29 (87.9)	17 (94.4)	0.45

Laterality of the LE intervention with respect to the transplanted kidney

**Figure 2:**
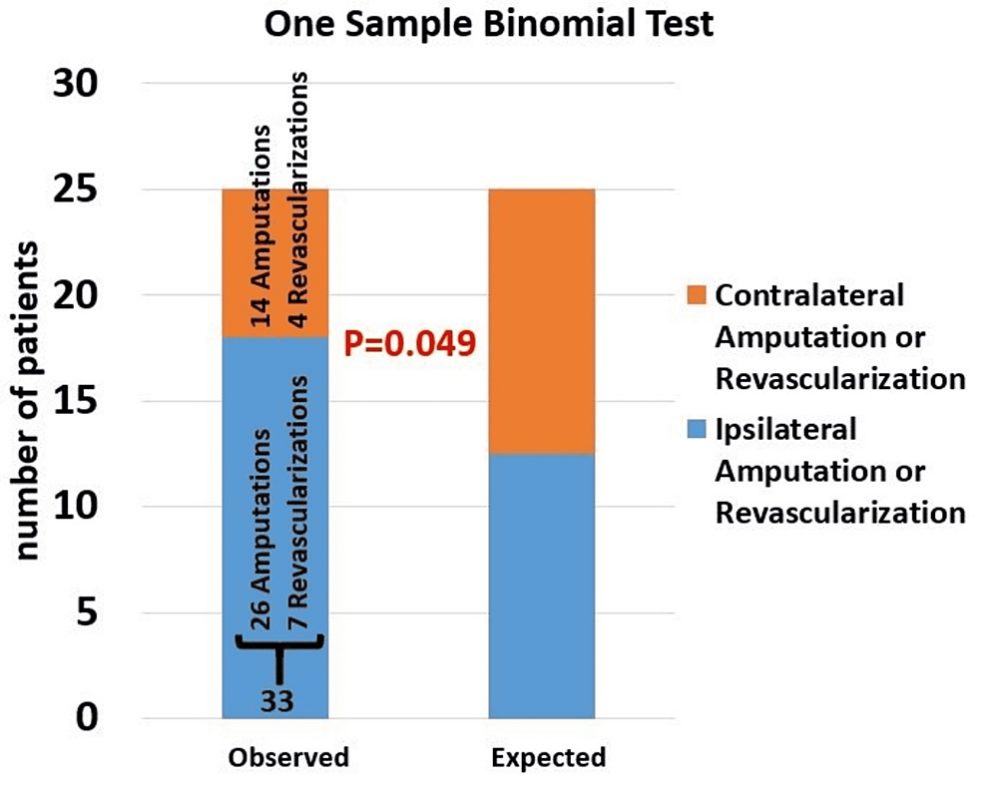
Comparison between ipsilateral and contralateral amputations and revascularizations using the one sample binomial test

A total of 33 patients (65%) underwent a LE intervention for ischemia due to PAD ipsilateral to the transplanted kidney. Of these, seven patients underwent revascularization (three bypasses and four endovascular procedures) as a first intervention following the transplant, while 26 patients underwent major amputations. On the other hand, 18 patients (35%) underwent LE PAD interventions (14 major amputations, one bypass, one endovascular, and two hybrid) contralateral to the transplanted kidney. Most of the patients had interventions for their infrapopliteal artery (78%; n = 39), while others had interventions on the superficial femoral artery (10%), popliteal artery (4%), common femoral artery (4%), and iliac artery (4%). No significant difference was seen between the ipsilateral and contralateral groups when comparing the type or location of intervention performed (P = 0.225 and P = 0.211, respectively). A total of six patients had diagnosis of pre-transplant PAD. However, this was not significantly different between the ipsilateral and contralateral groups (12.5% vs 11.2%; P = 0.88). There was no significant difference in the baseline ankle brachial indices done before the vascular procedure between the ipsilateral and contralateral groups (0.18 ± 0.16 and 0.21 ± 0.14, respectively; P = 0.64). Using the one-sample binomial test, which assumes a similar distribution between the unilateral and contralateral interventions groups, the incidence of the two interventions in our study sample (33 ipsilateral and 18 contralateral) was significantly different (P = 0.049, Figure 2). The mean time from kidney transplantation to an intervention was 52 ± 43 months for the ipsilateral group and 41 ± 32 months for the contralateral group (P = 0.33).

## Discussion

In this study, we showed that kidney transplant patients who required subsequent LE amputation or revascularization were more likely to have an ipsilateral intervention than a contralateral intervention. The time from kidney transplantation to required LE intervention did not differ between groups. There's a paucity of data on the incidence of CLTI after kidney transplantation. In this cohort of patients who received kidney transplantation, the incidence of CLTI was 2.6%.

Chronic kidney disease is a significant risk factor for the development of PAD, with an incidence as high as 30%. The incidence of non-traumatic LE amputations among ESRD patients is thought to be 10 times higher than in the general population, even when controlled for diabetes [16,17]. ESRD is a significant poor prognostic factor for limb salvage and overall survival in patients with limb ischemia [18]. Survival in ESRD patients after open revascularization at one and three years is only 60% and 18%, respectively [3-5].

Kidney transplantation remains a mainstay of treatment for patients with ESRD. Although patients with a functioning kidney transplant have increased overall survival compared to patients who are on dialysis, kidney transplant recipients are at increased risk of developing atherosclerosis secondary to immunosuppressive therapy [19,20]. Moreover, risk factors commonly present in transplant waitlist patients such as hyperglycemia, hypertension, and hyperlipidemia, are worsened by immunosuppressive therapy and newly developed cardiometabolic disorders post-transplantation [19,20]. PAD remains a very common post-transplantation complication, with an overall estimated prevalence of 15% [21]. However, the relative risk of PAD in transplant recipients is about 23% lower than in patients who are on a waiting list [21]. Nonetheless, PAD has been shown to be an independent risk factor for allograft failure and death among kidney transplant patients [14,22,23]. One prospective cohort study showed that kidney transplant patients with PAD had three times higher risk of graft loss as compared to transplant patients without PAD [24]. This implies the significance of early detection and management of PAD along with its associated modifiable risk factors. Current clinical practice guidelines recommend treatment of symptomatic LE PAD prior to transplantation. 

Stenosis and thrombosis of the transplanted renal artery at the anastomotic site with the recipient iliac artery are among the most common vascular complications following kidney transplantation [25]. In our patient population, none of the patients who progressed to CLTI had an anastomotic complication. Development of LE ischemia in kidney transplant patients with PAD is postulated to be secondary to progression of underlying atherosclerotic disease [8]. In a retrospective study performed by Northcutt et al., of the 219 patients who underwent successful kidney transplantation, eight patients with known history of PAD developed new symptoms of ischemia within a year following their transplant. Because these symptoms developed late after transplantation, these ischemic complications were attributed to progression of underlying PAD [9]. Although some studies have suggested that kidney transplantation to the iliac artery does not significantly reduce distal limb flow in adults, acute limb ischemia due to the steal phenomenon by a graft kidney has been reported in pediatric patients [9,11,15,26].

The clinical significance of this steal phenomenon in adult patients with PAD has been a main concern of transplant and vascular surgeons in the post-transplantation evaluation, since it could lead to subsequent deterioration of the ischemic limb [20]. It has been postulated that around 700 mL of blood gets drawn per minute from the iliac arteries after kidney transplantation [27]. In a normal LE arterial system, autoregulation ensures that distal blood flow is not significantly compromised. However, this might not be fully possible in patients with PAD, which might lead to supply-demand imbalance and subsequent ischemia to the legs [27,28]. Few studies have explored this “blood steal” phenomenon. A small pilot study of 18 patients measured postoperative blood flow in the ipsilateral and contralateral legs after kidney transplantation and showed no significant difference between the two legs [28]. This study, however, was limited by its small sample size, measurement of blood flow at rest, and having had only one patient with a confirmed PAD diagnosis. We postulate that the ipsilateral transplanted kidney may contribute to development of CLTI because the kidney steals blood at the iliac level, and we recommend that future research should focus on evaluating the blood flow dynamics in the iliac arteries at rest and during exercise in kidney transplant patients with established PAD diagnosis.

Early detection and management of PAD after transplantation and reduction of potentially modifiable risk factors would lower graft malfunction, death, and PAD-associated complications in kidney transplant patients.

Limitations

This analysis was performed retrospectively and is subject to the inherent limitations of retrospective studies. Data collection was performed through chart reviews and registries. The study was performed within a single center and has a limited number of subjects who met the inclusion criteria. Finally, this study was performed at a quaternary care center. Many patients were referred to our center for the sole purpose of undergoing kidney transplantation and did not receive further follow-up, which might have underestimated the incidence of CLTI.

## Conclusions

Many patients who receive a kidney transplant develop LE CLTI, and in our patient sample, we observed an incidence of 2.6%. In our population, kidney transplant patients who subsequently required LE surgical intervention were more likely to have it on the ipsilateral side of the kidney transplant. The etiology of this association is unclear, and further studies are needed to determine the etiology of this association. Kidney transplant patients should be monitored closely for the development of ischemic symptoms in the LE.
